# Locating Medical and Recreational Cannabis Outlets for Research Purposes: Online Methods and Observational Study

**DOI:** 10.2196/16853

**Published:** 2020-02-26

**Authors:** Eric R Pedersen, Caislin Firth, Jennifer Parker, Regina A Shih, Steven Davenport, Anthony Rodriguez, Michael S Dunbar, Lisa Kraus, Joan S Tucker, Elizabeth J D'Amico

**Affiliations:** 1 RAND Corporation Santa Monica, CA United States; 2 RAND Corporation Arlington, VA United States; 3 RAND Corporation Boston, MA United States; 4 RAND Corporation Pittsburgh, PA United States

**Keywords:** marijuana, cannabis, dispensaries, retailers, Los Angeles, tobacco

## Abstract

**Background:**

An increasing number of states have laws for the legal sale of recreational and medical cannabis out of brick-and-mortar storefront locations. Given the proliferation of cannabis outlets and their potential for impact on local economies, neighborhood structures, and individual patterns of cannabis use, it is essential to create practical and thorough methods to capture the location of such outlets for research purposes. However, methods used by researchers vary greatly between studies and often do not include important information about the retailer’s license status and storefront signage.

**Objective:**

The aim of this study was to find methods for locating and observing cannabis outlets in Los Angeles County after the period when recreational cannabis retailers were granted licenses and allowed to be open for business.

**Methods:**

The procedures included searches of online cannabis outlet databases, followed by methods to verify each outlet’s name, address, license information, and open status. These procedures, conducted solely online, resulted in a database of 531 outlets. To further verify each outlet’s information and collect signage data, we conducted direct observations of the 531 identified outlets.

**Results:**

We found that 80.9% (430/531) of these outlets were open for business, of which 37.6% (162/430) were licensed to sell cannabis. Unlicensed outlets were less likely to have signage indicating the store sold cannabis, such as a green cross, which was the most prevalent form of observed signage. Co-use of cannabis and tobacco/nicotine has been found to be a substantial health concern, and we observed that 40.6% (175/430) of cannabis outlets had a tobacco/nicotine outlet within sight of the cannabis outlet. Most (350/430, 81.4%) cannabis outlets were located within the City of Los Angeles, and these outlets were more likely to be licensed than outlets outside the city.

**Conclusions:**

The findings of this study suggest that online searches and observational methods are both necessary to best capture accurate and detailed information about cannabis outlets. The methods described here can be applied to other metropolitan areas to more accurately capture the availability of cannabis in an area.

## Introduction

### Background

A majority of states in the United States now have laws for legalized and decriminalized cannabis. As of October 2019, 33 states and the District of Columbia have passed medical cannabis laws, which grant access to residents enrolled in state medical cannabis programs, and 11 states and the District of Columbia have legalized the possession and sale of retail cannabis for adults aged 21 years and older. In many of these states, legal cannabis can be purchased for personal consumption from brick-and-mortar storefront locations (*cannabis outlets*), such as medical cannabis dispensaries and recreational cannabis retailers. Preliminary evidence suggests that cannabis outlet locations are associated with certain economic, neighborhood, and social environmental factors (eg, property and violent crimes, racial/ethnic population density, and parental physical abuse) [1-3], and proximity to cannabis outlets in one’s neighborhood is associated with personal use in both cross-sectional [4-6] and longitudinal studies [7,8] of adults and adolescents. However, findings are inconsistent across studies, which may be due, in part, to a lack of standardization in measuring access to cannabis outlets.

Unfortunately, there is no best practice to guide the measurement of access to cannabis outlets in legalized states, and the methods used by researchers to collect outlet location information vary greatly between studies. Most of this work has focused on medical cannabis dispensaries in California and Colorado [2,3,5,9-14] and on recreational cannabis retailers in Colorado and Washington [7,8]. With few exceptions, previous researchers describe the methods used for determining locations of these outlets in just a few sentences at most, which makes it difficult to determine the details and extensiveness of these procedures, while also making it impossible to replicate these methods for future work.

In addition, most studies use the official city, county, or state registries of cannabis outlets to determine locations and information on whether or not each outlet is open for business. However, these lists fail to capture the network of cannabis outlets that are unlicensed, but still operational, which are known to operate quite extensively throughout California [15]. Researchers have used internet-based methods, such as cannabis outlet search engines (eg, Weedmaps and Leafly), to locate unlicensed and licensed outlets [2,5], but these search engines often do not distinguish between licensed and unlicensed outlets. License status information is important as consumers may feel more comfortable purchasing cannabis from a legitimate retailer, but the potential prevalence of unlicensed retailers may make access to cannabis more available to those who may not want to travel to a licensed retailer.

It is also crucial to know about signage and storefront advertisements because without such details, it cannot be determined if an individual could tell whether the outlet sells cannabis or not. Yet, only one study to date has included signage information [6]. Researchers collected detailed storefront signage by reviewing all available images of medical cannabis dispensaries on the internet (eg, customer-uploaded pictures on Yelp, Google Maps images, and owner-posted pictures on Weedmaps); however, images of some storefronts could not be found, and some available images may have been outdated. In addition, there may have been other information around the storefront that indicated the outlet sold cannabis, which was not observable in online pictures alone, such as sidewalk signs, posters, murals, or billboards with clear cannabis references. Thus, although the study revealed important findings regarding the association between storefront signage and cannabis use by young adults, more nuanced information about signage is needed.

### This Study

This paper describes the methods that build on previous efforts by providing a detailed methodology that can be replicated in large metropolitan areas that have legalized the sale of cannabis. We selected Los Angeles County because of the densely populated area, racial/ethnic and economic diversity, recent proliferation of recreational cannabis outlets starting in January 2018 (after legalization for recreational sale and possession in November 2016), and accurate and comprehensive state- and city-level sources of licensed retailers. Similar to our previous work [16], we first conducted extensive internet searches for cannabis outlets in Los Angeles County to generate a database of outlets we believed to be currently open and operational. We verified information about license claims from the outlets’ online content using the newly updated directory of licensed cannabis outlets created and maintained by the California Bureau of Cannabis Control (BCC). Finally, knowing the limitations of using only internet-based searches of outlets signage from prior work [16], we followed observational procedures used by researchers in prior medical cannabis dispensary and vape shop work [2,17,18] to conduct in-person observations of storefronts and generate detailed information about the cannabis outlets. Such details about the cannabis outlet environment could help to provide an understanding of the impacts of specific characteristics of cannabis outlets on both youth and adult use.

## Methods

### Internet Database Searches and Cleaning Procedures

In December 2018, we extracted data from Weedmaps and Leafly on store name, address (including number, street, city, and ZIP code), phone number, license information, whether the store offered delivery, date the retailer created an account on the website, date of last update, store hours, and websites/social media sites for all cannabis outlets (ie, medical cannabis dispensaries and recreational cannabis retailers) within California. Our prior work indicated that using additional websites, such as Yelp, or other cannabis outlet databases, such as StickyGuide or Where’s Weed, provided very few additional open outlets outside of Weedmaps and Leafly alone [16]. At the time of our data extraction, Leafly only included verified licensed medical and/or recreational cannabis outlets, whereas Weedmaps included any medical and/or recreational outlets registered on their site, regardless of the license status. From earlier studies, the research team had developed a program to automatically navigate website contents to dispensary and retailer pages and extract key data (eg, store location) [16], but performing the present scrape required updating an earlier generation of code to accommodate changes in the websites’ front-end structures (eg, changes in menu options and data displays).

In general, this process of scraping store listings requires (step 1) a method or data source of identifying all store URLs in the area of study and (step 2) a method for iterating through URLs and extracting data fields from the HTML source (or for dynamic pages where content is also produced from non-HTML sources). For this study, (step 1) our Weedmaps scrape began with a list of store URLs on the website, whereas the Leafly scrape proceeded by entering ZIP codes into the dispensary search box (using a headless browser), thereby identifying Web links for each city page, providing a second set of links to iterate through looking for store URLs. For both methods, (step 2) once all URLs were gathered, HTML pages were iterated over to extract data.

After the data were obtained from each website source (N=198 on Leafly and N=1037 on Weedmaps), we combined files to remove duplicate outlets, dropped outlets outside of Los Angeles County (based on the 526 Los Angeles County ZIP codes), and conducted procedures developed in our prior work to verify store names and addresses [16]. Such procedures included verifying that addresses and store names for outlets featured on both Weedmaps and Leafly were consistent; reviewing outlet website and social media pages (eg, Facebook, Instagram, and Twitter); conducting Google and Yelp searches of the outlet name and address to verify information across multiple websites; and reading recent customer reviews on outlet websites, Google, and Yelp to determine if customers mentioned outlet name or address inconsistencies (eg, “I tried to go to this store and it wasn’t at the address posted online” and “This place is closed.”). These procedures, especially customer reviews, helped determine whether the outlet was currently operating and open for business. If store name, address, and open/closed status could not be determined after exhausting all internet-based methods, we called stores to verify this information. All cleaning procedures and license verification procedures were conducted in February 2019.

### License Verification Procedures

We extracted content from each of the Weedmaps and Leafy websites to indicate whether the outlet had a state license (medical, recreational, or both) to sell cannabis. We also reviewed the content on each website (eg, *About* section of the outlet’s profile) to determine if the store indicated they had a license. We verified cannabis business licenses for all outlets by reviewing the City of Los Angeles Department of Cannabis Regulation–authorized retail business database, an online registry of licensed cannabis retailers in the City of Los Angeles, and the License Search Tool on the California BCC website, which is an online tool to verify license numbers and lists all the medical cannabis dispensaries and recreational cannabis retailers in the state that have licenses. This latter tool was necessary to verify licenses for cannabis outlets within Los Angeles County that were outside of Los Angeles City and not captured by the city registry.

### Observational Procedures: Outlet Site Visits

The final step was to verify each cannabis outlet’s information by conducting site visits. We developed procedures for driving to each cannabis outlet and collecting information that could be observed within a 360-degree view (side to side and up and down) from the front of the store. Using Google Maps, we planned for 1 observer to drive to each of the identified outlets within the 4750 square miles of Los Angeles County (4058 of which is land) during the open hours found online to (1) verify the address and name of the outlet, (2) verify that the outlet was open for business, (3) record the signage included on storefronts (eg, signs on doors and products that could be visually observed inside the store from outside), (4) record other information related to the outlet or that referenced cannabis in the area (ie, content on billboards, sidewalk signs, posters, and murals; camera on site; and security guard outside), (5) identify other stores in the immediate area that sold cannabis (ie, other medical dispensaries and recreational retailers), and (6) identify other stores in the immediate area that sold electronic nicotine devices (eg, vape pens, electronic cigarettes, and Juice USB Lighting) and/or other nicotine and tobacco products (ie, specialty vape shops or smoke shops, grocery stores, convenience stores, and liquor stores). Identifying stores that sold tobacco/nicotine products was important, given the prevalence of young people’s reports of tobacco/nicotine and cannabis co-use, which is linked with heavier use of both substances and mental and physical health problems [19-25]. The same observer took a photo at the address of each cannabis outlet. These observations were completed by 3 research staff observers during April 2019 and took approximately 230 hours (divided by 3 observers) to complete. See Multimedia Appendix 1 for the data collection instrument used by the observers in the study.

### Descriptive Statistics

We conducted descriptive statistics and used the Pearson chi-square test at the .05 level of significance to detect differences in cannabis outlet characteristics between licensed and unlicensed retailers.

### Map of Cannabis Outlets

We used the results of the observational study to map all cannabis outlets currently operating in Los Angeles County. Using ArcMap (v.10.7.1; Environment Systems Research Institute, INC, Redlands, California [26]), we geocoded each cannabis outlet and mapped both licensed and unlicensed cannabis outlets within Los Angeles County to their latitude and longitude.

## Results

### Open Status of Cannabis Outlets

From the original data extraction of cannabis outlets on Weedmaps and Leafly, 531 outlets were identified in Los Angeles County and determined to be open through online procedures alone. Observers visited each of these 531 outlets and determined that 80 (15.0%) were clearly closed, typically because another business had moved in, there was a *for rent* sign, or the building was vacant and the outlet was nowhere else in site. Of the remaining 451 outlets, 28 (6.2%) could not be identified as open because of no clear storefront signage and no indicator that a dispensary or retailer (or business of any kind) was located at the address. Our research team reviewed images of outlet storefronts (eg, a building with graffiti, a chained and locked garage door, and windows boarded up) and attempted to determine if the outlet was open/closed by comparing previous Google Maps images of the outlets with the more recent photo taken by observers, looking at Yelp or Google reviews that might indicate the business had closed, exploring whether social media sites and websites had been removed, and calling available phone numbers and determining if the line was disconnected or we were told that the business had shut down. Of the 28 unclear dispensaries, 7 (25%) were determined to be open through these procedures, whereas the remainder were determined to be closed. Thus, our database for analyses described below contained 430 verified open cannabis outlets in Los Angeles County.

### Type of Cannabis Outlet and License Information

Table 1 shows the number and percentage of outlets that claimed to sell only medical cannabis or recreational cannabis or both as well as whether or not we were able to verify their license status. Of the 430 outlets, 166 (38.6%) claimed to have licenses to sell medical and/or recreational cannabis. Most outlets that claimed to have a license online were verified as having a license: 95.9% (142/148) of the outlets that claimed to have both a medical and a recreational license were verified, and 92% (12/13) of the outlets that claimed to have a recreational license only were verified. Very few retailers claimed to only have a license to sell medical cannabis (5/430, 1.0% of all outlets), and 60% (3/5) of these retailers were verified. Five outlets were verified as having a license, although they did not claim in any online sources we reviewed to have either a medical or a recreational license. Across all 430 open outlets, 268 (62.3%) outlets were found to be unlicensed retailers. This included 9/268 (3.3%) outlets that claimed online to have a license but were found to not have one; 252/268 (94.0%) outlets that did not have a license and did not claim to have one; and 7/268 (2.6%) outlets that had undeterminable license status based on all available information using online, phone, and observational methods.

**Table 1:** Cannabis outlets by license claim status and by verification of license categories (Table includes cell counts and column percentages).

Online claims about licensure by outlet type	Verified license for medical only, recreational only, and recreational and medical outlets (n=162)	Verified unlicensed (n=268)
Claimed only a medical license (n=5)^a^, n (%)	3 (60)	2 (40)
Claimed only a recreational license (n=13), n (%)	12 (92)	1 (8)
Claimed both medical and recreational licenses (n=148), n (%)	142 (95.9)	6 (4.1)
Did not claim any license (n=257), n (%)	5 (2.0)^b^	252 (98.0)
Unclear from available information (n=7), n (%)	N/A^c^	7^d^ (100.0)

^a^One retailer that claimed to only have a medical license was found to have a verified recreational license.

^b^This includes five stores that did not claim a recreational or medical license and had a verified recreational and medical license. This table presents mutually exclusive categories.

^c^N/A: not applicable.

^d^Seven stores that we were not able to verify license information for were categorized as “unlicensed.”

### Cannabis Outlet Signage

We subjectively coded the signage information from the coding sheet used by the observers, where there were 22 indicators of signage, to help determine if it was clear that the outlet sold cannabis (see Multimedia Appendix 1). We did this for storefronts and sidewalk signs, billboards, posters, and murals in the immediate area. Table 2 displays the signage indicators across all formats (storefronts, sidewalk signs, billboards, posters, and murals) by outlet license status. Unlicensed outlets were less likely to have clear signage than licensed ones, with 36.2% (97/268) of unlicensed outlets having no clear signage compared with 13.6% (22/162) of licensed outlets (χ^2^_1_=25.8; *P*<.001)

**Table 2:** Indicator of clear signage across all formats (storefronts, sidewalk signs, billboard, posters, and murals) and by license status (Table includes cell counts and column percentages).

Signage format	Total verified license (N=162)		Unlicensed outlets (n=268), n (%)	Total all outlets (N=430), n (%)
	Licensed recreational only or in combination with medical (n=160)^a^, n (%)	Licensed medical only (n=2), n (%)		
No clear signage	22 (13.8)	0 (0)	97 (36.2)	119 (27.7)
Green cross only	30 (18.8)	0 (0)	91 (34.0)	121 (28.1)
Green cross and other clear signage^b^	67 (41.9)	2 (100)	44 (16.4)	113 (26.3)
Other clear signage^b^ only (no green cross)	41 (25.6)	0 (0)	36 (13.4)	77 (17.9)

^a^Column percentages are over 100% due to precision in rounding.

^b^Other clear signage refers to the nongreen cross indicators that cannabis was sold inside the outlet.

#### Storefronts

Of the 430 outlets, 311 (72.3%) had signage indicating that they sold cannabis, and 119 (27.6%) either had no signage at all or signage that was not clearly indicative that the store sold cannabis (eg, storefronts with an open sign and tinted windows but no signage related to what was sold inside). The most consistently reported type of clear signage was a green cross, with 51.6% (222/430) of outlets including this type of storefront sign. Of those outlets with a green cross, 51.3% (114/222) solely had a green cross that identified the outlet as selling cannabis. Table 3 shows the number of outlets that featured each type of clear cannabis signage on storefronts.

**Table 3:** Storefront signage for the 430 cannabis outlets.

Signage format	Outlets with clear storefront signage (not mutually exclusive), n (%)
Green cross	222 (51.6)
Cannabis leaf	71 (16.5)
Other cannabis-related words (eg, “420,” “THC,” “sativa,” “dispensary”)	48 (11.2)
Indicator that outlet sells recreational cannabis	45 (10.5)
Abundance of green color^a^	40 (9.3)
Indicator that outlets sells medical cannabis	29 (6.7)
“Cannabis” or “weed”	26 (6.0)
“Pre-ICO”^b^	24 (5.6)
“Prop-D compliant” or “Prop-64 compliant”^c^	24 (5.6)
Green caduceus symbol	15 (3.5)
“CAP” (eg, “$25 CAP”)^d^	12 (2.8)

^a^Abundance of green color on the outlets was determined to be a clear indicator as it was typically in the context of other clear signs, most often a green cross. Only four outlets had an abundance of green color without other clear signage indicators.

^b^“Pre-ICO“ refers to medical marijuana dispensaries that were operating before September 14th 2007, when the Medical Marijuana Interim Control Ordinance (ICO) was established.

^c^Prop-D refers to tax paying medical dispensaries that were prioritized to receive a retail license (over new cannabis retailer applicants) after January 1st 2018. Prop-64 refers to the Adult Use of Marijuana Act passed by California voters in November 2016.

^d^“CAP” refers to the highest amount a consumer would pay for the top-shelf cannabis flower at the outlet.

#### Sidewalk Signs

Approximately one-fourth (106/430, 24.6%) of the outlets had a sidewalk sign outside: 50 licensed outlets had sidewalk signs, and 56 unlicensed retailers had sidewalk signs. The most common forms of sidewalk signage were a green cross (66/106, 62.3%), followed by the name of the outlet that indicated it sold cannabis (41/106, 38.7%), a cannabis plant leaf (17/106, 16.0%), and other wording or symbols that indicated the outlet sold cannabis (17/106, 16.0%).

#### Billboards

Only 13 outlets had a billboard advertising their specific outlet within the immediate area (13/430, 3.0%). Of these 13 billboards, 8 (62%) featured a green cross, 2 (15%) featured the word *cannabis*, 1 (8%) featured the name of the outlet with an abundance of green color, and 2 (15%) featured cannabis imagery with references to specific brands or products (eg, *green ghost*). For 14 outlets, there were billboards for another cannabis outlet within the immediate area of the targeted outlet.

#### Posters and Murals

Only 13 outlets had any posters or murals outside (13/430; 3.0%). Most of these posters or murals either contained the name of the store (6/13, 46%) or a green cross (6/13, 46%). Of 13 outlets, 2 posters (15%) had both a green cross and the store name, and 3 posters (23%) had the word *cannabis*.

### Other Characteristics of Outlets

#### Security

Most outlets (384/430, 89.5%) had a security camera located outside the storefront, and 15.8% (68/430) of outlets had a security guard(s) outside. Licensed outlets were more likely to have a security guard (41/162, 25.3%) compared with unlicensed outlets (27/268, 10.1%; χ^2^_1_=17.6; *P<*.001). Furthermore, licensed outlets were more likely to have a security camera (152/162, 93.8%) compared with unlicensed outlets (232/268; 86.6%; χ^2^_1_=5.6; *P=*.02).

#### Vape and Tobacco Shops

We also coded stores in the immediate visible area of each outlet to determine if surrounding stores sold tobacco and/or nicotine products. These included specialty tobacco and vape stores, liquor stores, and convenience stores. A total of 40.9% (175/428) of cannabis outlets had stores in the immediate visible area that sold tobacco/nicotine products. Approximately one-fourth (49/175, 28.0%) of these cannabis outlets had specialty vape shops nearby, and 84.0% (147/175) of the outlets had other tobacco/nicotine retailers nearby (20/175, 11.4% had both specialties vape shops and other tobacco/nicotine retailers nearby). Licensed outlets were less likely to have a tobacco/nicotine retailer nearby (54/162, 33.3%) compared with unlicensed shops (122/268, 45.5%; χ^2^_1_=6.2; *P=*.01). It should be noted that we looked for storefront tobacco and/or nicotine product advertisements on the cannabis outlets themselves and found that none of the outlets contained such advertisements.

#### Cannabis Outlets Across Los Angeles County

**Figure 1:**
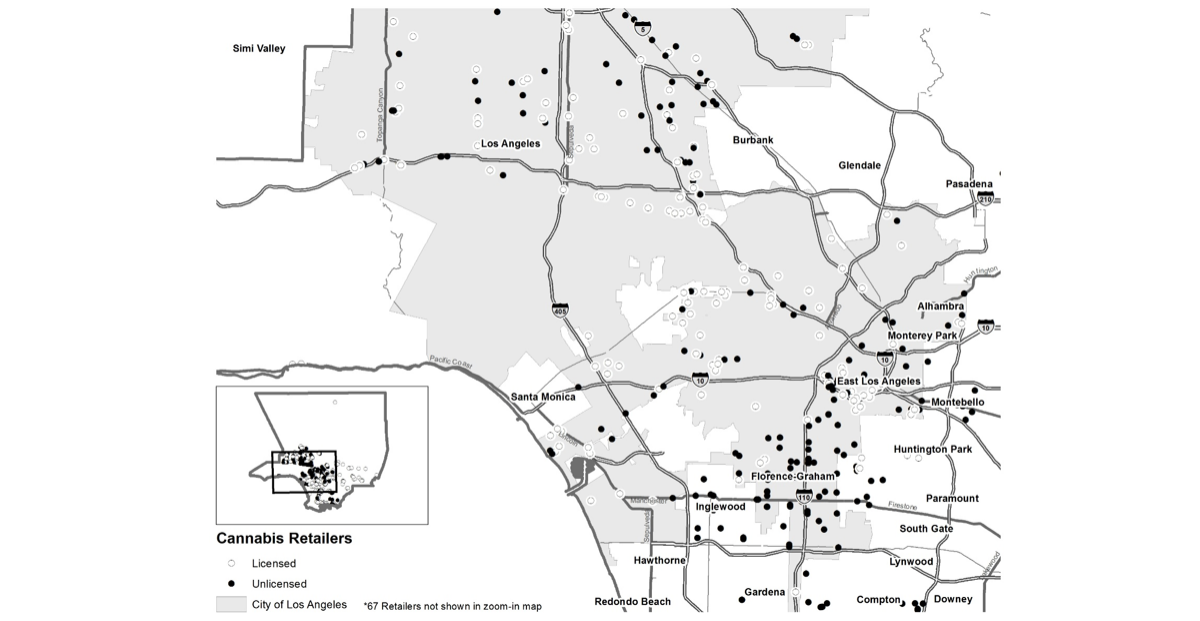
Map of Cannabis outlets in Los Angeles County. (Map is current as of April 2019 when the direct observations were completed.)

Of the 430 cannabis outlets, 49 (11.4%) had another cannabis outlet in the immediate visible area, and 350 (81.4%) cannabis outlets within Los Angeles County were in the City of Los Angeles, which consists of 503 square miles (469 square miles of land). Outlets were not distributed evenly throughout the city and tended to cluster near downtown Los Angeles (see Figure 1). Less than half (142/350, 40.6%) of the cannabis outlets within the City of Los Angeles were licensed. However, outlets within the city were significantly more likely to be licensed than outlets in other areas of the County (14/80, 18% of outlets outside of the city; χ^2^_1_=17.0; *P*<.001). Furthermore, there appeared to be spatial patterning in the locations of licensed outlets, such that outlets in central Los Angeles or in key commercial areas were more likely to be licensed, as evident by the shading in Figure 1.

## Discussion

### Summary of Findings

There is a need for standardized, comprehensive, and practical methods to locate cannabis outlets. These methods can help researchers design studies to better understand the effects of cannabis dispensaries and retailers on neighborhood quality and determine cannabis-related societal and public health outcomes. In this study, we described online and observational methods to create a point-in-time snapshot of open cannabis outlets with brick-and-mortar storefronts in Los Angeles County and outline procedures for researchers to verify license information, capture signage, and document other pertinent environmental characteristics of cannabis outlets. Building off our prior work using internet-based methods alone [16], we identified 531 cannabis outlets operating in Los Angeles County. However, after conducting observational site visits, it was determined that only 80.9% (430/531) of these outlets were operational. Although the observations were conducted 4 months after the internet-only search was conducted, had we not conducted site visits, we would have overestimated the number of operating cannabis outlets by about 19.0% (101/531 originally identified were closed). Thus, a combination of online searches and observational methods appears important to best capture accurate and detailed information about cannabis outlets.

The observational procedures required our members of the research team to visit 531 cannabis storefronts and record characteristics of the storefronts and the immediate environment. Although this endeavor was time consuming (approximately 234 total hours across 3 observers), it was feasible because many of the cannabis outlets in Los Angeles County were clustered in central Los Angeles (see Figure 1). Other work in this area has shown that cannabis outlets cluster in areas of low socioeconomic status in Washington State and Colorado [27,28], and prior work has also shown this to be the case for medical cannabis dispensaries in California [3,29]. This is important as the clustering of cannabis outlets may disproportionately expose certain neighborhoods and area residents to cannabis retailers. Retailers may also choose to locate their businesses in areas where they know there are a lot of established consumers. It should also be noted that observational methods alone, such as *ground truthing*, where observers would drive every street in an area to locate targeted retailers (eg, locating vape shops [18]), would be unfeasible, given there are 4751 square miles in Los Angeles (85% of which are land), and also that many cannabis outlets identified from the online sources were unrecognizable during observations as outlets that sold cannabis (ie, 27.7% had no signage indicating the outlet sold cannabis). This confirmed that observational procedures alone may be insufficient and that a combination of observational procedures with online searches is needed.

In addition to using registries of licensed cannabis outlets hosted by city- and state-level regulatory agencies, the use of online cannabis outlet finder websites was essential to gather information about both licensed and unlicensed outlets. We found that the majority (62.3%) of cannabis outlets in Los Angeles County were unlicensed, and these unlicensed cannabis outlets were less likely to have signage indicating the outlet sold cannabis or to have security guards and cameras outside. Leafly removed all unlicensed cannabis outlets in California from its website in March 2018 to comply with the California BCC’s regulations of advertising online; thus, we used Weedmaps to identify unlicensed cannabis outlets for this study. The California BCC has pressed Weedmaps to remove unlicensed cannabis outlets in California; however, as of October 2019, the website still advertised unlicensed outlets. Should Weedmaps comply with the California BCC, locating unlicensed cannabis outlets may prove more difficult in California. However, unlicensed outlets located in other states would still be available on the website in other states unless these states follow the California BBC’s efforts and pursue this action with Weedmaps as well. In addition, other websites exist that may still offer searchable features for unlicensed outlets (eg, StickyGuide, Where’s Weed, and Yelp). In some cases, future research may be able to use Web archiving services (eg, the Internet Archive Wayback Machine) to collect historic information from some online resources that have since been removed or modified; however, archives for cannabis outlet registry sites such as Weedmaps and Leafly are generally not available from the Wayback Machine. Researchers interested in preserving these data for future research use may do so by running website scraping programs now, to be regularly rerun and maintained going forward, producing a proprietary database of historical data.

The inclusion of signage information in our database represents a major innovation as it enables researchers to examine the effects that variability in storefront signage on cannabis retailers may have on population health outcomes. Given that more than one-fourth of the 430 cannabis outlets had no signage indicating that the outlet sold cannabis, these discreet storefronts may go unnoticed. A green cross was the main indicator of signage, but a substantial number of outlets featured a cannabis plant leaf or advertised through the actual word *cannabis*. A clear indication that a store sells cannabis is imperative for determining the effects that emerging commercial cannabis markets may have on cannabis use behaviors. In other substance use areas, for example, researchers have found a positive association between visible tobacco advertisements and sale of cigarettes to youth under the legal smoking age in Massachusetts [30]. We found a similar signage effect in our cross-sectional medical cannabis dispensary work conducted in Los Angeles County in 2017, whereby signage in front of medical cannabis dispensaries was strongly associated with young adult use [6].

### Limitations

The methods described here are not without limitations that researchers should consider when constructing a database of cannabis outlets for their own studies in Los Angeles and in other areas. It should be noted that the timeline between data extraction from the online databases (Weedmaps and Leafly) and observations of the outlets was approximately 3 to 4 months. This time was needed to develop procedures and implement methods, but ideally, time between data extraction and observations would be shorter, as there may have been cannabis outlets in the County that opened during that window as well as outlets open at the time of the online database searches that closed by the time observations were conducted. Indeed, 101 of the field visits were to an outlet that was no longer operating. It is unclear if these outlets would have been operating if we had conducted observations immediately after (or during) cleaning and verification procedures. When we replicate these methods for future work, we will be able to significantly shorten the timeline as methods have now been established and tested. Second, conducting outlet observations was time and labor intensive. Given budgetary restrictions, only one research staff member coded each cannabis outlet. In future data collection efforts, we will improve the reliability of cannabis outlet coding by having 2 observers double code 10% of all outlets, estimate interrater reliability with a Cohen kappa coefficient, and have observers reach consensus on coding discrepancies before the remaining outlets are surveyed. Replication of these methods in other jurisdictions will allow researchers to establish longitudinal databases of cannabis outlets to better capture the duration of exposure that residents have to cannabis retailers. We encourage researchers using these methods to attempt to expedite their procedures as well. One way to do this could involve multimodal mobile surveillance systems, which have been used to collect data on tobacco retailers and involve the use of text messages, email, GPS technologies, photographs, and phone-based interactive voice response using mobile phones [31].

Another limitation of this work is that the methods used here may not accurately determine access to cannabis outlets via delivery services. During observations, the research team coded whether storefronts, sidewalk signs, billboards, posters, and murals contained any information about whether the outlet itself offered delivery (see Multimedia Appendix 1). Only two billboards, one sidewalk sign, one poster, and none of the storefronts mentioned delivery or had an advertisement for a third-party cannabis delivery service. A factor that was not measured in this study was the availability of third-party cannabis delivery services (eg, Eaze) that pick up cannabis from established brick-and-mortar dispensaries and retailers and deliver it to residents. This makes cannabis more accessible to individuals that may live far from cannabis outlets or in municipalities that do not permit brick-and-mortar storefronts. Information about delivery services offered by the outlet itself is helpful, but studies that seek to examine how access to cannabis is affected by the emergence of cannabis outlets may need to incorporate information about the areas served by these delivery services.

Finally, although we used Yelp, Google, and social media websites to help verify information about the cannabis outlets after our initial extraction of data from Weedmaps and Leafly, we did not expand our initial searches beyond the two online cannabis outlet databases. One reason for this was because prior work had shown that using other cannabis outlet databases and Yelp yielded only a trivial number of additional cannabis outlets not obtained from Weedmaps or Leafly alone [16]. An additional reason is that it is difficult to determine which search terms to use on generalized search engines, such as Yelp and Google. Outlets rarely include the words *cannabis*, *marijuana*, or *pot* in their names, and the outlets that self-identify as selling cannabis would likely be the licensed outlets that we already obtained via Weedmaps and Leafly. However, it is possible that the methods described in this study missed unlicensed outlets in Los Angeles that either had no online presence or advertised on different websites.

### Conclusions

This study is the first to detail methods for collecting crucial information about cannabis outlets in a large metropolitan area with both licensed and unlicensed medical cannabis dispensaries and recreational cannabis outlets. The findings provide important lessons learned about how well online and observational methods work for brick-and-mortar retailers, which are the predominant mode of cannabis sales in legalized states. If delivery services become more popular over time, future research should validate methods for searching for availability of delivery services and variability in individuals’ purchasing behaviors in stores vs online delivery.

## Appendix

Multimedia Appendix 1 Observational coding sheet.

## Abbreviations

BCC: Bureau of Cannabis Control
