# Supplementary material for: Locating Medical and Recreational Cannabis Outlets for Research Purposes: Online Methods and Observational Study
Source: J Med Internet Res. 2020 Feb 26;22(2):e16853. doi: 10.2196/16853 (PMC7066509; doi:10.2196/16853)
Supplement: Multimedia Appendix 1 [file jmir_v22i2e16853_app1.docx]

**APPENDIX: OBSERVATION CODING SHEET**

**NAME**

Store Name: [FILL FROM SAMPLE FILE]

1. Same
2. Different (Enter Store Name: SPECIFY **DIFFNAME**)

**ADDRESS**

Store Address: [FILL FROM SAMPLE FILE]

1. Same
2. Different (Enter Address: [SPECIFY])
3. Store is not there or anywhere in site of address in database

[IF ADDRESS=3, ASK. ELSE GO TO OPEN.]

**Inegaddr-ADDRESS2**

Is this address now a:

- 1. New business (not marijuana-related)
  2. New marijuana outlet (SPECIFY **inegaddr-newoutletsp**)
  3. Vacant building

Other (SPECIFY **inegaddr-ADDRESS2_other**)

[GO TO COMMENTS]

**OPEN**

Is this a business that is still open? (not necessary right now)

1. Yes
2. Unclear (Describe: [SPECIFY])
3. No (Describe: [SPECIFY])

**PHOTO**

INTERVIEWER: TAKE A PHOTO OF THE STOREFRONT

**STOREFRONT**

Does the store name appear on the storefront?

1. Yes
2. No

**STOREFRONT1**

Is there anything on the storefront that indicates the store may sell marijuana (including an indicator in the store name):

1. Yes
2. No

[IF STOREFRONT1=1, ASK STOREFRONT2. ELSE GO TO GUARD]

**STOREFRONT2**

Check the box for each of these that is visible **On the Storefront (including in the store name)**:

|  |  | **Yes** |
| --- | --- | --- |
| a | Abundance of green color |  |
| b | Words “cannabis,” “weed,” “pot,” or “marijuana” (including in store name) |  |
| c | Words indicating store sells medical marijuana (e.g., medical marijuana card) |  |
| d | Words indicating store sells recreational marijuana (e.g., now offering recreational, no medical card required, 21+, adult use) |  |
| e | Other words referencing marijuana or marijuana products (e.g., bong, reefer, 420, THC, CBD)  [SPECIFY] |  |
| f | Pot leaf |  |
| g | Green cross |  |
| h | Green caduceus medical symbol (if altered, like 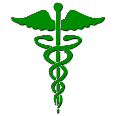with pot leaves instead of wings, include under “other”) |  |
| i | Paraphernalia visible from the street (e.g., bongs in window) |  |
| j | License number |  |
| k | Prop-D Compliant/Friendly |  |
| l | Prop 64 Compliant/Friendly |  |
| m | Pre-ICO |  |
| n | Prop 215 Compliant/Friendly |  |
| o | The word “legal” |  |
| p | “Cash only” |  |
| q | The words “dispensary,” “canna,” or “green” |  |
| r | Mention delivery or have an advertisement for marijuana delivery service (e.g., Eaze) |  |
| s | A brand/strain of marijuana sold at the store |  |
| t | Indicator of what the CAP is (e.g., 25 CAP, $35 CAP) |  |
| u | Promotion for paraphernalia sold in the store (e.g., bongs, papers, vaping devices) |  |
| v | “First-time patient” or FTP deals/gifts |  |
| w | Anything nicotine/tobacco-related (e.g., e-cigs, cigarettes, juul, mods, vaping devices for nicotine) |  |
| x | Other (anything marijuana-related; e.g., “sativa,” “edibles,” “indica,” “wax”)  [SPECIFY] |  |

**GUARD**

Is there a security guard outside?

1. Yes
2. No

**CAMERA**

Is there a visible security camera?

1. Yes
2. No

**SIDEWALK**

Are there any sidewalk signs referring to this store or to marijuana in a 360 view of the store?

1. Yes
2. No

[IF SIDEWALK=YES, ASK SIDEWALK1. ELSE, GO TO POSTER.]

**SIDEWALK1**

[PROGRAMMER: THIS IS A CHECK ALL THAT APPLY. INTS MUST BE ABLE TO CHECK ALL OR SOME OF BOXES A-W.]

Check the box for each of these that is visible **On Sidewalk Signs**:

|  |  | **Yes** |
| --- | --- | --- |
| a | Name of store |  |
| b | Abundance of green color |  |
| c | Words “cannabis,” “weed,” “pot,” or “marijuana” (including in store name) |  |
| d | Words indicating store sells medical marijuana (e.g., medical marijuana card) |  |
| e | Words indicating store sells recreational marijuana (e.g., now offering recreational, no medical card required, 21+, adult use) |  |
| f | Other words referencing marijuana or marijuana products (e.g., bong, reefer, 420, THC, CBD)  [SPECIFY] |  |
| g | Pot leaf |  |
| h | Green cross |  |
| i | Green caduceus medical symbol (if altered, like 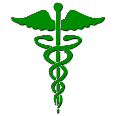with pot leaves instead of wings, include under “other”) |  |
| j | Paraphernalia visible from the street (e.g., bongs in window) |  |
| k | License number |  |
| l | Prop-D Compliant/Friendly |  |
| m | Prop 64 Compliant/Friendly |  |
| n | Pre-ICO |  |
| o | Prop 215 Compliant/Friendly |  |
| p | The word “legal” |  |
| q | “Cash only” |  |
| r | The words “dispensary,” “canna,” or “green” |  |
| s | Mention delivery or have an advertisement for marijuana delivery service (e.g., Eaze) |  |
| t | A brand/strain of marijuana sold at the store |  |
| U | Indicator of what the CAP is (e.g., 25 CAP, $35 CAP) |  |
| V | Promotion for paraphernalia sold in the store (e.g., bongs, papers, vaping devices) |  |
| W | “First-time patient” or FTP deals/gifts |  |
| X | Anything nicotine/tobacco-related (e.g., e-cigs, cigarettes, juul, mods, vaping devices for nicotine) |  |
| y | Other (anything marijuana-related; e.g., “sativa,” “edibles,” “indica,” “wax”)  [SPECIFY] |  |

**POSTERS**

Are there any posters or murals referring to this store or marijuana in a 360 view of the store (not including on the storefront)?

1. Yes
2. No

[IF POSTERS=1, ASK POSTERS1. ELSE, GO TO OTHER SIGNAGE]

**POSTERS1**

[PROGRAMMER: THIS IS A CHECK ALL THAT APPLY. INTS MUST BE ABLE TO CHECK ALL OR SOME OF BOXES A-W.]

Check the box for each of these that is visible **On Posters or Murals**:

|  |  | **Yes** |
| --- | --- | --- |
| a | Name of store |  |
| b | Abundance of green color |  |
| c | Words “cannabis,” “weed,” “pot,” or “marijuana” (including in store name) |  |
| d | Words indicating store sells medical marijuana (e.g., medical marijuana card) |  |
| e | Words indicating store sells recreational marijuana (e.g., now offering recreational, no medical card required, 21+, adult use) |  |
| f | Other words referencing marijuana or marijuana products (e.g., bong, reefer, 420, THC, CBD)  [SPECIFY] |  |
| g | Pot leaf |  |
| h | Green cross |  |
| i | Green caduceus medical symbol (if altered, like 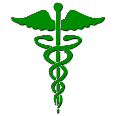with pot leaves instead of wings, include under “other”) |  |
| j | Paraphernalia visible from the street (e.g., bongs in window) |  |
| k | License number |  |
| l | Prop-D Compliant/Friendly |  |
| m | Prop 64 Compliant/Friendly |  |
| n | Pre-ICO |  |
| o | Prop 215 Compliant/Friendly |  |
| p | The word “legal” |  |
| q | “Cash only” |  |
| r | The words “dispensary,” “canna,” or “green” |  |
| s | Mention delivery or have an advertisement for marijuana delivery service (e.g., Eaze) |  |
| t | A brand/strain of marijuana sold at the store |  |
| u | Indicator of what the CAP is (e.g., 25 CAP, $35 CAP) |  |
| v | Promotion for paraphernalia sold in the store (e.g., bongs, papers, vaping devices) |  |
| w | “First-time patient” or FTP deals/gifts |  |
| x | Anything nicotine/tobacco-related (e.g., e-cigs, cigarettes, juul, mods, vaping devices for nicotine) |  |
| y | Other (anything marijuana-related; e.g., “sativa,” “edibles,” “indica,” “wax”)  [SPECIFY] |  |

**OTHER SIGNAGE**

Are there any other marijuana-related advertisements such as flyers on telephone poles or advertisements at bus stops (that you have not already documented)?

1. Yes ([SPECIFY**]**)
2. No

**OTHER STORES**

Turn in a circle to observe a full 360 degrees. Do you see any of the following?

**VAPE**

Specialty vape shop or smoke shop (store that appears to sell vape juice, devices, and accessories)? May also sell cigarettes, pipes, joint wraps, and other tobacco/nicotine paraphernalia.

1. Yes
2. No

[IF VAPE=1, ASK VAPEa-d on same screen. ELSE GO TO TOBACCO]

**VAPE1**

|  |  | **Yes** |  |
| --- | --- | --- | --- |
| a | Any visible signs for e-cigs or other vaping products |  |  |
| b | Words “vape,” “vapor,” “vaping,” “e-cig,” “e-cigarette,” “juul,” “juuling,” “mods” |  |  |
| c | Any visible signs that store sells cigarettes, pipes, joint wraps, and other tobacco/nicotine paraphernalia |  |  |

**VAPEd**

What is the name of the store?

**TOBACCO**

Other tobacco/nicotine retailer (convenience stores, grocery stores, liquor stores)?

1. Yes
2. No

[IF TOBACCO=1, ASK TOBACCOa-d on same screen. ELSE GO TO OUTLETS]

**TOBACCO1**

|  |  | **Yes** |  |
| --- | --- | --- | --- |
| a | Any visible signs for e-cigs or other vaping products |  |  |
| b | Words “vape,” “vapor,” “vaping,” “e-cig,” “e-cigarette,” “juul,” “juuling,” “mods” |  |  |
| c | Any visible signs that store sells cigarettes, pipes, joint wraps, and other tobacco/nicotine paraphernalia |  |  |

**TOBACCOd**

What is the name of the store?

**OUTLETS**

Another marijuana outlet?

1. Yes
2. No

[IF OUTLETS=1, ASK OUTLETSa on same screen. ELSE GO TO BILLBOARDS]

**OUTLETSa**

What is the name of the store?

**BILLBOARDS**

Billboard for this marijuana outlet?

1. Yes
2. No

[IF BILLBOARDS=1, ASK BILLBOARDSa-c on same screen. ELSE GO TO BILLBOARDS1]

**bboard-BILLBOARDSa**

|  |  | **Yes** |  |
| --- | --- | --- | --- |
| a | Mentions selling medical marijuana |  |  |
| b | Mentions selling recreational marijuana |  |  |

**bboard-BILLBOARDSc**

Describe the billboard:

**BILLBOARDS1**

Billboard for another marijuana outlet?

1. Yes
2. No

[IF BILLBOARDS1=1, ASK BILLBOARDS1a-c on same screen. ELSE GO TO BILLBOARDS2]

**bboard1-BILLBOARDS1a**

|  |  | **Yes** |  |
| --- | --- | --- | --- |
| a | Mentions selling medical marijuana |  |  |
| b | Mentions selling recreational marijuana |  |  |

**bboard1-BILLBOARDS1c**

Describe the billboard:

**BILLBOARDS2**

Advertisements for marijuana delivery service (e.g. Eaze)?

1. Yes
2. No

**BILLBOARDS3**

Billboard for non-outlet specific marijuana content (e.g. a brand of marijuana)?

1. Yes
2. No

**COMMENTS**

Please enter any additional comments:

**END**
